# LncRNA HOTAIR contributes Taxol-resistance of hepatocellular carcinoma cells via activating AKT phosphorylation by down-regulating miR-34a

**DOI:** 10.1042/BSR20201627

**Published:** 2020-07-30

**Authors:** Yunfei Duan, Jing Chen, Yu Yang, Zhen Qu, Yunjie Lu, Donglin Sun

**Affiliations:** Department of Hepatopancreatobiliary Surgery, The Third Affiliated Hospital of Soochow University, Changzhou 213161, China

**Keywords:** apoptosis, hepatocellular carcinoma, Taxol-resistance

## Abstract

Drug resistance of Taxol leads to the treatment failure in hepatocellular carcinoma (HCC). LncRNA HOTAIR have drawn increasing attention in various diseases; its function and mechanism in Taxol-resistance in HCC remain unclear. In the present study, the two Taxol resistant HCC cell lines (HepG2/Taxol and SMMC7721/Taxol) were induced. The qRT-PCR data exhibited that over-expressed HOTAIR as well as low-expressed miR-34a were founded in HepG2/Taxol and SMMC7721/Taxol cells. HOTAIR knockdown suppresses proliferation, invasion and promotes apoptosis of in HepG2/Taxol and SMMC7721/Taxol cells through up-regulating miR-34a by MTT assay, transwell invasion assays and flow cytometry, while down-regulation of miR-34a had an opposite effect on reversing Taxol resistance. Cleaved caspase-3 and Bax were significantly up-regulated by si-HOTAIR transfection, while Bcl-2 level exhibited opposite trend. Besides, HOTAIR knockdown impaired Taxol-resistance in HCC by accommodating Akt phosphorylation and Wnt/β-catenin signaling via interacting with miR-34a. The present study may afford a valuable target for treating Taxol-resistance in HCC.

## Introduction

Hepatocellular carcinoma (HCC) is one of the main types of human primary liver cancer, and its mortality rate is second highest in the world among various cancer [1]. In the last few decades, the treatment of HCC has been gradually improved, but its mortality rate is still high [2]. Only up to 30% of the patients are suitable for radical resection or transplantation, and systemic chemotherapy is demanded for advanced HCC patients [3]. Yet chemoresistance and failures are often developed during treatments [4]. The mechanisms of chemoresistance of the tumor cells are complicated, including disorder of the critical signaling pathways, changes of the targets of anticancer drugs, the increased drug efflux and disordered expression of RNA, DNA or proteins [5,6]. Taxol was the common drug for HCC chemotherapy. However, drug resistance of Taxol leads to its less efficiency. As a result, it was urgent to discover the underlying molecular mechanisms to up-regulate Tax-sensitivity.

With the development of genomics and transcriptomics, plentiful noncoding RNAs (ncRNAs) have been demonstrated with regulative ability in cellular and physiologic process [7,8]. Long noncoding RNAs (lncRNAs) are a class of noncoding RNAs with transcripts greater than 200 nucleotides in length [9]. Through a series of studies, it has been found that lncRNAs are essential in life activities, and there is tremendous research value, especially with tumors [10–12]. HOX transcript antisense RNA (HOTAIR) is the first found IncRNA gene with trans-acting [13]. Lots of scientific researches have confirmed that HOTAIR is over-expressed in a variety of solid tumors, and the abnormal increase of HOTAIR is closely related to the infinite proliferation of tumor cells, growth promotion, angiogenesis, migration and metastasis [14,15]. MicroRNAs (miRNAs), a category of about 22-nucleotide noncoding RNAs, regulating gene transcription with translation suppression or target mRNAs recession [16]. The interactions between LncRNAs and miRNAs have been proved roundly [17].

Since the discovery of HOTAIR in 2007, it has received a lot of attention [18]. In 2010, HOTAIR was found to be closely related to the regulation of histone modifications [19]. Many studies have confirmed that HOTAIR affects the occurrence and development metastasis and prognosis of diverse cancers. For example, HOTAIR promotes osteosarcoma development by sponging miR-217 and targeting ZEB1, HOTAIR faciliates gastric cancer progression via miR-217-GPC5 axis, and HOTAIR regulates the development of non-small cell lung cancer through miR-217/DACH1 signaling pathway [20–23]. However, there are no intensive studies on the mechanism of HOTAIR in the Tax-resistance of hepatocellular carcinoma, which deserves in-depth research.

Additionally, the over-activation of AKT kinase signaling pathway plays a key role in resistance of hepatocellular carcinoma, which is either indirectly through the activation of intersecting oncogenic pathways or directly through PI3 kinase, somatic mutation of PTEN, or AKT itself, finally boost tumor survival, growth, and progression [24–26]. PTEN overexpression and PI3K inhibitors in PTEN-null cells have demonstrated the reversal of drug resistance [27–29]. However, the possible association of AKT activation and HOTAIR in Taxol-resistance of hepatocellular carcinoma have not been investigated.

We conducted the present study to investigate a role of HOTAIR in Taxol-resistance of hepatocellular cancer cells: Taxol-resistant HepG2 and Taxol-resistant SMMC7721. The results showed that HOTAIR and its binding target miR-34a were unusually expressed in Taxol-resistant hepatoma cells. Besides, low-expressed HOTAIR suppressed cell invasion, enhanced Taxol-induced apoptosis, and inhibited Akt phosphorylation and Wnt/β-catenin signaling pathways by up-regulating miR-34a, so as to reverse Taxol-resistance in hepatoma cells. Taken together, it was suggested that HOTAIR may be a promising novel target for Taxol-resistance in HCC treatment.

## Materials and methods

### Cell lines and resistance induction

We obtained human HCC cell line HepG2 and SMMC-7721 from the Cell Bank of the Institute of Biochemistry and Cell Biology (Shanghai, China) in 2009 and maintained in DMEM (Sigma-Aldrich, St. Louis, Missouri, U.S.A.). These cell lines contained 10% fetal bovine serum (FBS) (Hyclone, Logan, Utah, U.S.A.) and were placed at 37°C in 5% CO_2_. Based on the previous study, Taxol-resistant cells were selected from sensitive by stepwise increases in taxol concentrations from 50 to 250 nM till there were no dead cells. The resistant cells have been maintained in DMEM containing 100nM Taxol and 10% FBS. The resistant index (RI) was caluculated by IC50 of Taxol in Taxol-resistant cells / IC50 of Taxol in parental cells.

### Cell transfection

HOTAIR siRNA, miR-34a inhibitor or their corresponding negative controls (NCs) were obtained from GenePharma Co., Ltd. (Shanghai, China) and transfected into the cells with Lipofectamine 2000 (Invitrogen, Carlsbad, CA, U.S.A.).

### MTT assay

Cell viability was tested by the MTT assay to evaluate the Taxol sensitivity of normal HepG2 and SMMC7721 cells and Taxol resisted HepG2 and SMMC7721 cells. Briefly, 48-h after transfection, the cells were resuspended in complete medium, 100 μl was added to each well in a 96-well plate at a density of 1 × 10^4^ cells/well. They were co-incubated with different concentrations of Taxol (0, 2, 20, 40, 80, 100 μM) for another 48 h. Subsequently, 20 μl of MTT solution (5 mg/ml) was added to each well and cultured for another 4 h. The medium was removed and 100 μl of DMSO was added. The optical density was read at 490 nm (Bio Rad, Hercules, California, U.S.A.). The results were produced and calculated; all assays were carried out in triplicate.

### Real-time quantitative PCR

We used Trizol reagent (Invitrogen, Carlsbad, CA, U.S.A.) to extract total RNA from the tissue samples and cells. We conducted Real-time quantitative PCR (abbreviated as qPCR) in SYBR Green PCR Master Mix (Roche Diagnostic GmbH, Mannheim, Germany) with a total volume of 20 μl. We conducted all the reactions in duplicate. The 2^−ΔΔCT^ method was applied for normalization of mRNA levels to GAPDH mRNA levels. Primers used are shown as listed: HOTAIR forward: 5′-CCAG TTCT CAGG CGAG AGCC-3′ and reverse: 5′-TTTATATTCAGGACATGTAA-3′; miR-34a forward: 5′-CGGTATCATTTGGCAGTGTC T-3′ and reverse: 5′-GTGC AGGGTCCGAGGT AT-3′. β-actin forward: 5′-GATGAGATTGGCATGGCTTT-3′ and reverse: 5′-GTCACCTTCACCGTTCCAGT-3′. Each experiment was conducted triplets.

### Luciferase activity assay

Wild type (WT) or mutant (MUT) HOTAIR-bound miR-34a was inserted into pmirloGLO dual luciferase small RNA targeting expression vector (Promega, Madison, WI, U.S.A.) to form a reporter vector (HOTAIR/ WT or HOTAIR/MUT). Cells were then transfected with HOTAIR/WT or HOTAIR/ MUT and/or miR-34a mimics or miR-NC for 48 h. The dual luciferase reporter assay system provided by Promege (Madison, WI, U.S.A.) was utilized to determine luciferase activity.

### Cell invasion assay

HepG2/Taxol and SMMC7721/Taxol cells were seeded in a matrix gel-coated upper chamber (BD Bioscience) in an amount of 4 × 10^5^. The lower chamber was filled with medium containing 10% FBS, the cells were invaded into the filter for 24 h, then the cells at the top of the filter were removed, and the bottom cells were fixed in 4% paraformaldehyde and stained with crystal violet. The field of view was observed using an inverted microscope and counted (Olympus, Tokyo, Japan).

### Cell apoptosis

HepG2/Taxol and SMMC7721/Taxol cells and that transfected with si-NC or si-HOTAIR were processed with/without Taxol and anti-miR-34a for 48 h. Cells were collected for resuspending in binding buffer. A FITC-Annexin V-propidium iodide (PI) Apoptosis Detection Kit (Beyotime, Nanjing, China) was applied. The FACScan flow cytometer (BD Biosciences, CA, U.S.A.) was utilized to analyze apoptosis ratio.

### Western blotting

Western blotting was conducted based on previously reported methods. Lysis buffer containing protease inhibitors (Promega, Madison, WI) was used for the extraction of total protein. The protein concentration was detected by the BCA method. Cell lysates (100 μg) were separated on a gradient SDS-PAGE gel, then transferred to a PVDF membrane and then in a blocking solution (5% skim milk) in TBST buffer (10 mM Tris-HCl, pH 8.0, 150 mM NaCl and 0.1% Tween 20) for 1 h at room temperature to blocked non-specific binding. Subsequently, the films were then incubated with primary antibodies overnight at 4°C. Thereafter, the membranes were washed three times using TBST buffer and incubated with secondary antibodies for another 2 h at room temperature. After another washes, proteins were detected with ECL reagent. Here, β-actin acted as an internal reference and band intensity was analyzed by ImageJ software.

### Statistical analysis

GraphpadPrism7.0 (GraphPad Software, U.S.A.) was applied for statistical calculations. The evaluation of the differences between two groups was conducted using the Student’s *t*-test. And one-way analysis of variance (abbreviated as ANOVA) was employed to analyze the comparison of the means greater than or equal to three groups. Data were shown as the mean ± SD. *P*<0.05 was defined as statistically significant.

## Results

### Establishment of Taxol-resistant HepG2 and SMMC7721 hepatocellular cancer cells

**Figure 1:**
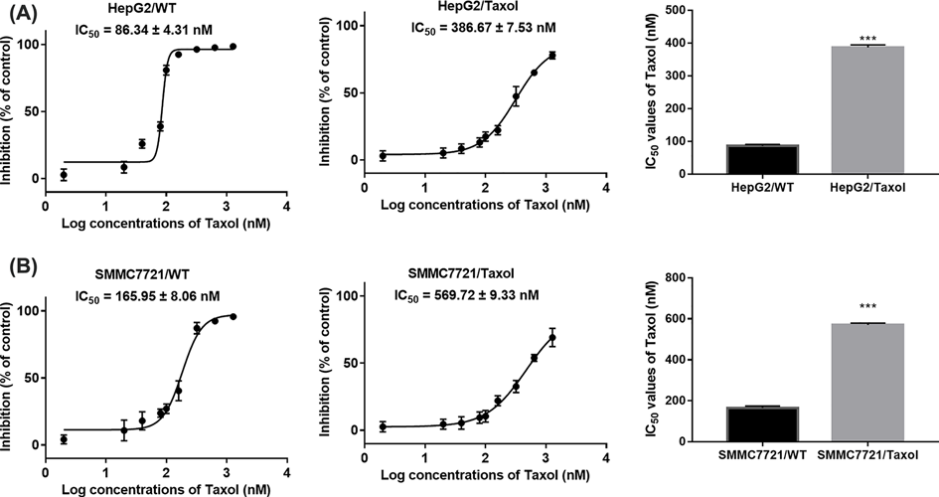
The successful establishment of Taxol-resistant HepG2 and SMMC7721 hepatocellular cancer cells (**A**) Inhibition rates and IC_50_ values of Taxol in HepG2/WT cells or HepG2/Taxol cells. (**B**) Inhibition rates and IC_50_ values of Taxol in SMMC7721/WT cells or SMMC7721/Taxol cells. ***P<0.001 vs. respective wild-type cells.

HCC is the second leading cause of cancer-related deaths in the word, and chemoresistance and failures are often developed in the patients’ treatments. To study the mechanisms of chemo-resistance, we developed two Taxol resistant hepatocellular cancer cells: HepG2/Taxol and SMMC7721/Taxol. To test the taxol resistance ability of these cells, we cultured wide type and Taxol resistant HepG2 or SMMC7721 with indicated concentrations of Taxol (from 2 to 1280 nM). To determine the cell viability under Taxol cultured environment, MTT assay were performed. As showed in Figure 1A,B, the 50% inhibitory concentration (IC_50_) in HepG2 and SMMC7721 wide type cells were 86.34 ± 4.31 nM and 386.67 ± 7.53 nM (RI of 4.48), while in HepG2 and SMMC7721 Taxol resistant type were 165.95 ± 8.06 nM and 569.72 ± 9.33 nM (RI of 3.43), separately. The results indicated that these two Taxol resistant types of HepG2 and SMMC7721 were successfully established.

### HOTAIR and miR-34a expressions are unusual in HepG2/Taxol and SMMC7721/Taxol cells

**Figure 2:**
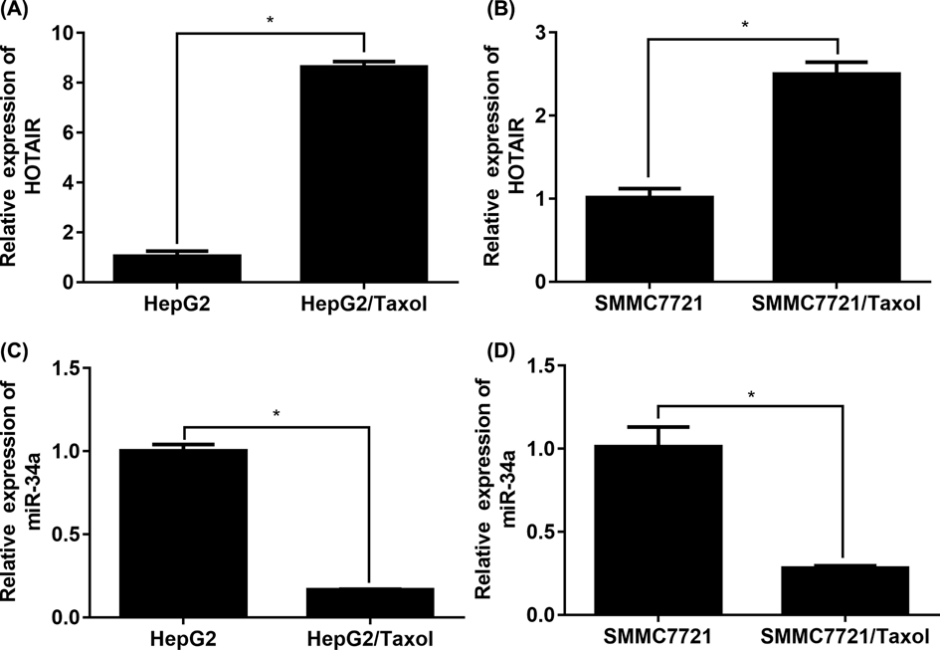
Expression of HOTAIR and miR-34a are unusual in taxol resistant HepG2 and SMMC7721 cells (**A**) HOTAIR expression in HepG2/WT cells or HepG2/Taxol cells. (**B**) HOTAIR expression in SMMC7721/WT cells or SMMC7721/Taxol cells. (**C**) miR-34a expression in HepG2/WT cells or HepG2/Taxol cells. (**D**) miR-34a expression in SMMC7721/WT cells or SMMC7721/Taxol cells. The data were present as means ± SD of three independent experiments. *P<0.05 vs. respective wide type cells.

The mRNA expression of HOTAIR and miR-34a in normal/Taxol resistant HepG2 and SMMC7721 cells were detected firstly. Based on the data in Figure 2A,B, HOTAIR was highly more up-regulated in Taxol resistant cells than that in normal sensitive cells. Moreover, miR-34a level in Taxol resistant cells was significantly reduced compared with normal sensitive cells (Figure 2C,D). The findings suggested that the unusual expression of HOTAIR could play a role in Taxol-resistance in HCC cells.

### miR-34a is a binding target of HOTAIR

**Figure 3:**
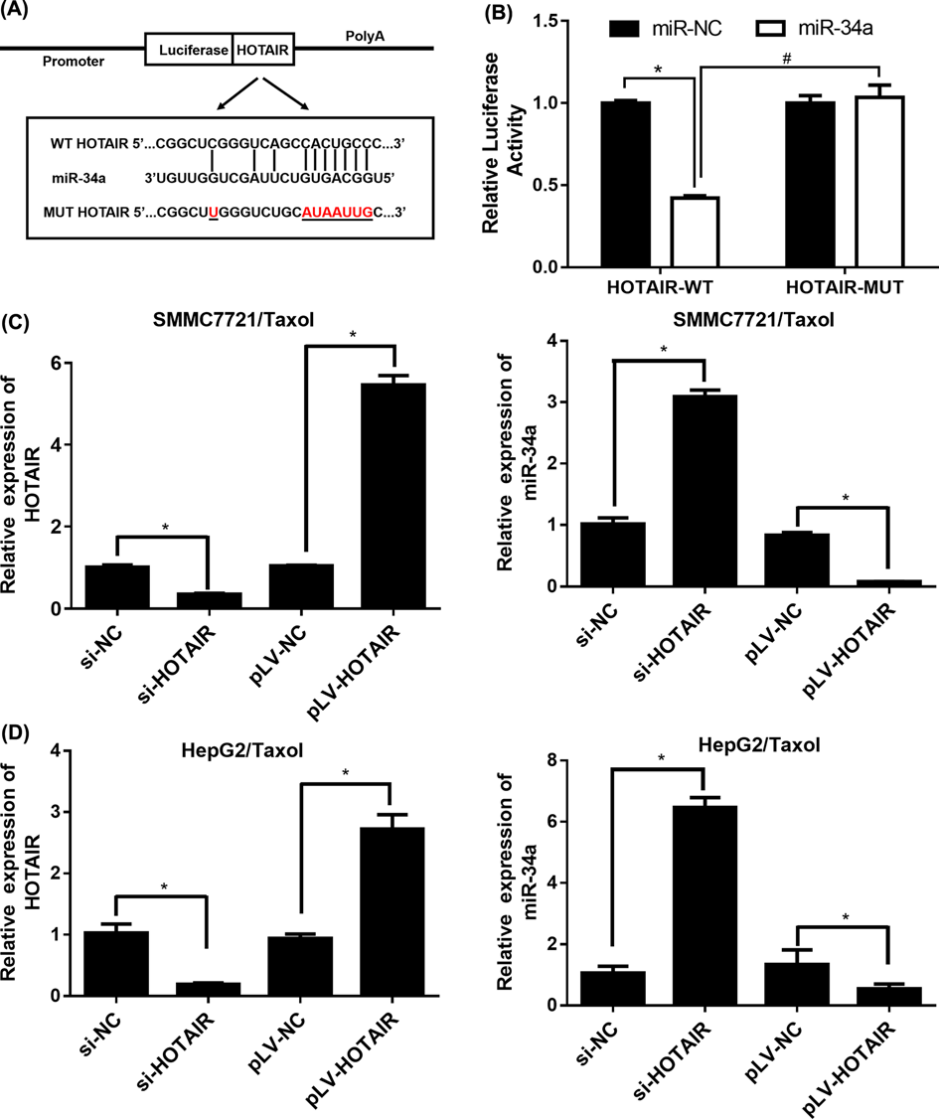
miR-34a is a binding target of HOTAIR (**A**) Binding region between miR-34a and HOTAIR and the luciferase reporter constructs containing HOTAIR/WT or HOTAIR/MUT sequence. (**B**) Relative luciferase activity of co-transfected into HepG2/Taxol cells with miR-34a. (**C**) Relative expression of HOTAIR and miR-34a in SMMC7721/Taxol cells. (**D**) Relative expression of HOTAIR and miR-34a in HepG2/Taxol cells. The data were present as means ± SD of three independent experiments. *P<0.05, ^#^*P*<0.05.

Further mechanism of HOTAIR reversing the Taxol-resistance in HCC was studied. For seeking the target gene of HOTAIR, miRanda, LncBase and Starbase databases were utilized. The intersection results of prediction targets in the three databases indicated that miR-34a was identified as a factor that was negative regulated by HOTAIR, that was demonstrated with luciferase reporter assay in HepG2/Taxol cells (Figure 3A). The luciferase activity in Figure 3B was significantly reduced in the HOTAIR/WT group, while there was no change in luciferase activity in the HOTAIR/MUT group. Therefore, it was suggested that miR-34a was the binding target of HOTAIR.

The transfection efficiency of si-NC or si-HOTAIR in both HepG2/Taxol and SMMC7721/Taxol cells were determined by qRT-PCR. Figure 3C,D revealed the expression of HOTAIR was remarkably down-regulated in cells and miR-34 expression had negative correlation with HOTAIR, which suggested that the expression of HOTAIR in HCC cells would be suppressed through the direct binding between miR-34a and WT HOTAIR.

### HOTAIR knockdown suppresses proliferation and invasion of HepG2/Taxol and SMMC7721/Taxol cells through up-regulating miR-34a

**Figure 4:**
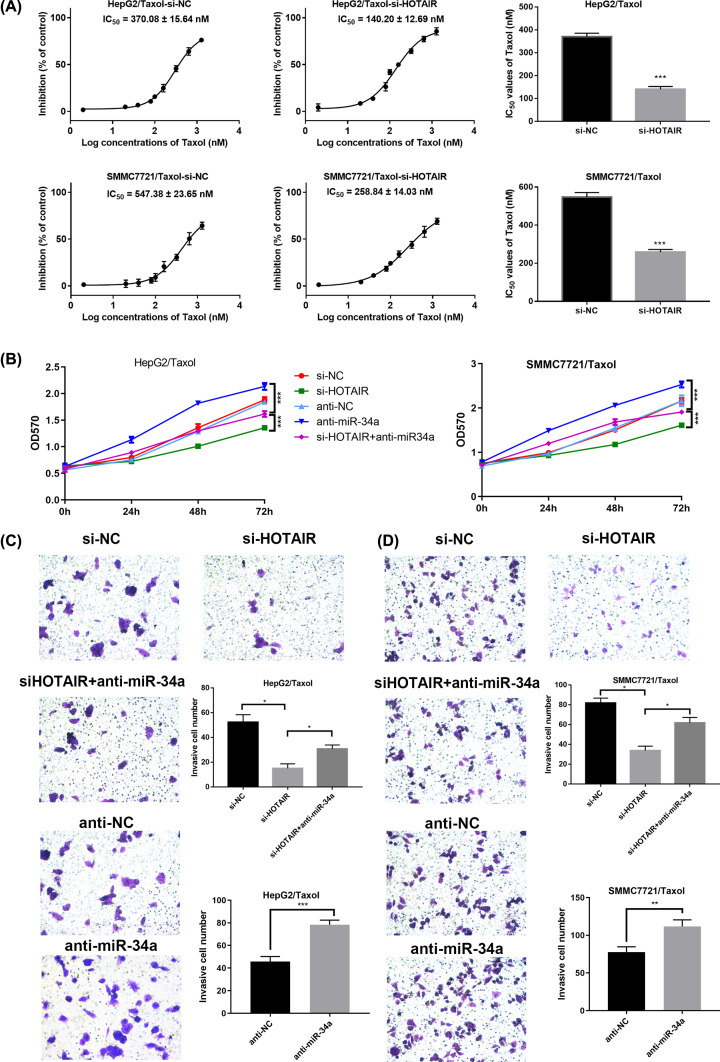
HOTAIR knockdown suppresses proliferation and invasion of HepG2/Taxol and SMMC7721/Taxol cells through up-regulating miR-34a (**A**) Proliferation rates and IC50 values of HepG2/Taxol or SMMC7721/Taxol after transfections. (**B**) The proliferation curves after transfections in HepG2/Taxol or SMMC7721/Taxol cells. Typical images of the invasion assays: (**C**) HepG2/Taxol cells treated with si-NC; si-HOTAIR; si-HOTAIR+anti-miR-34a; anti-NC; anti-miR-34a; (**D**) SMMC7721/Taxol cells treated with si-NC; si-HOTAIR; si-HOTAIR+anti-miR-34a; anti-NC; anti-miR-34a. The means of three independent biological replicates are shown; error bars indicate the SD; *P<0.05, **P<0.01, ***P<0.001.

To explore the role of LncRNA HOTAIR and miR-34a in the proliferation and invasion of HepG2/Taxol and SMMC7721/Taxol *in vitro*, the MTT and transwell invasion assay intervened with LncRNA HOTAIR siRNAs and anti-miR-34a was demonstrated. As shown in Figure 4A, si-HOTAIR transfection significantly inhibited the proliferation of HepG2/Taxol and SMMC7721/Taxol cells, while anti-miR-34a transfection promotes the cell proliferation, weakening the reversing-resistance effect of si-HOTAIR transfection. In addition, the IC50 values of cells after transfected with si-HOTAIR were significantly lower than that with si-NC transfection. The MTT results in Figure 4B exhibited that si-HOTAIR significantly inhibited the proliferation of resistant cells, while anti-miR-34a weakened this effect. The 24-h invasion of HepG2/Taxol and SMMC7721/Taxol cells in the si-HOTAIR group was significantly decreased compared with the si-NC group. At the same time, the 24-h invasion of HepG2/Taxol and SMMC7721/Taxol cells in anti-miR-34a group was significantly up-regulated compared with anti-NC group, and anti-miR-34a transfection significantly impaired anti-invasion effect of si-HOTAIR (Figure 4C,D). These results revealed that HOTAIR knockdown suppresses proliferation and invasion of HepG2/Taxol and SMMC7721/Taxol cells through up-regulating miR-34a.

### HOTAIR knockdown enhances apoptosis induced by Taxol in HepG2/Taxol and SMMC7721/Taxol cells via regulating miR-34a

**Figure 5:**
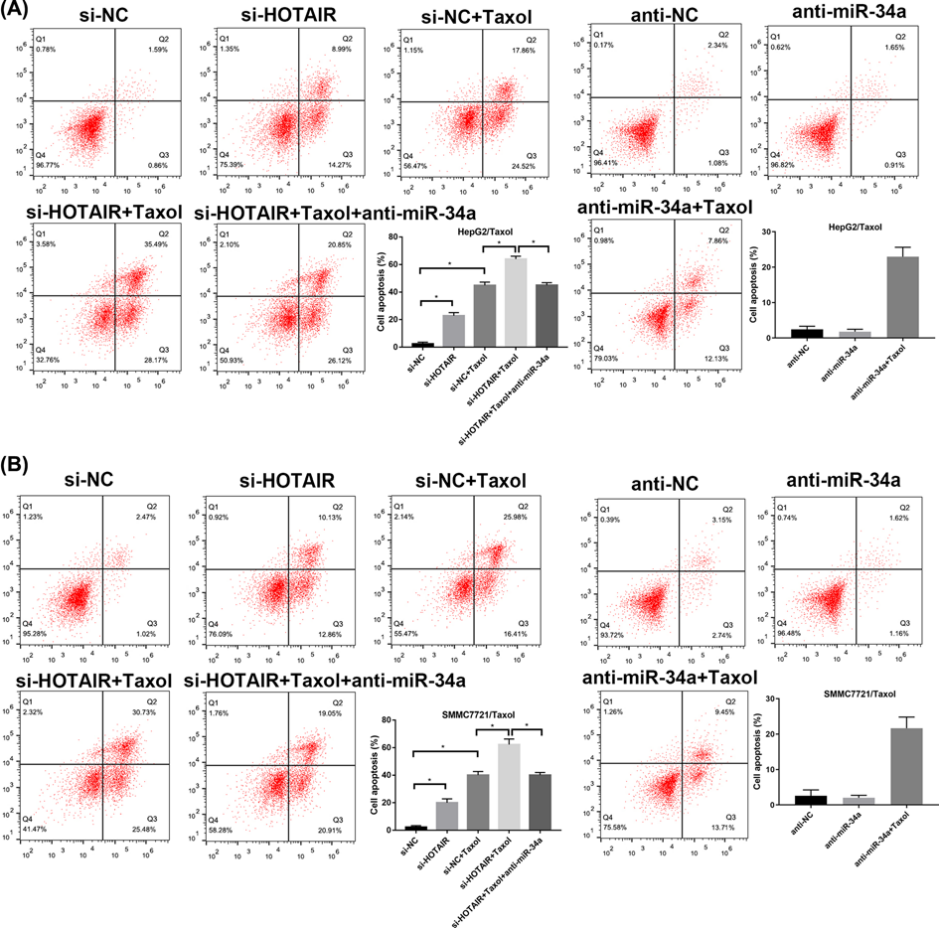
Effect of HOTAIR and miR-34a on apoptosis rates in HepG2/Taxol and SMMC7721/Taxol cells (**A**) HepG2/Taxol cells treated with si-NC, si-NC+Taxol, si-HOTAIR, si-HOTAIR +Taxol, si-HOTAIR+Taxol+anti-miR-34a, anti-NC, anti-miR-34a and anti-miR-34a+Taxol. (**B**) SMMC7721/Taxol cells treated with si-NC, si-NC+Taxol, si-HOTAIR, si- HOTAIR +Taxol, si- HOTAIR+Taxol+anti-miR-34a, anti-NC, anti-miR-34a and anti-miR-34a+Taxol. The means of three independent biological replicates are shown; error bars indicate the SD. *P<0.05 vs. different controls.

After 24-h transfection, 500 nM Taxol was added to the cells in specified groups and incubated for another 48 h for apoptosis determination by flow cytometry. As shown in Figure 5, the apoptosis rates were obviously elevated by si-HOTAIR transfection or/and Taxol treatment, and significantly reduced by anti-miR-34a transfection. Downregulation of HOTAIR strenthened the effect of Taxol. Besides, after transfection with anti-miR34a, the apoptosis-promoting effect caused by si-HOTAIR transfection or/and Taxol treatment was recovered (Figure 5).

**Figure 6:**
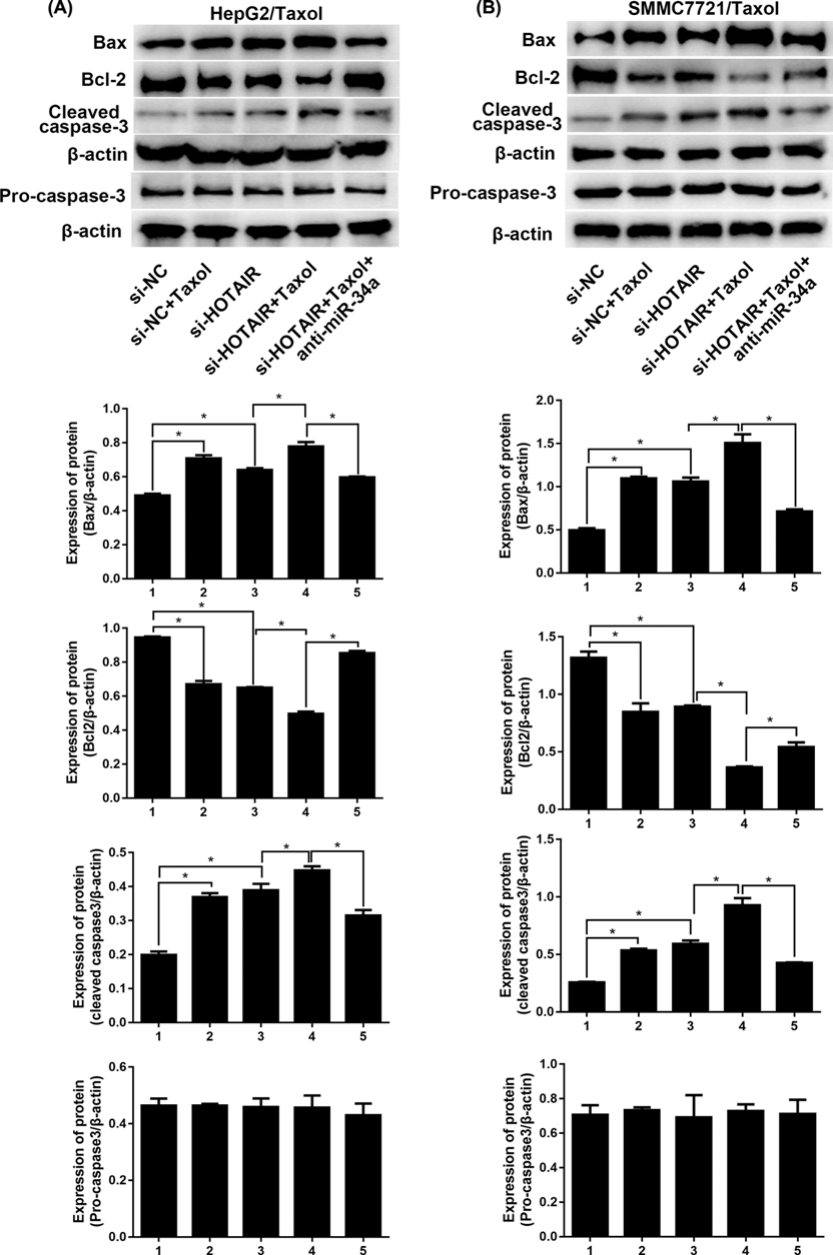
HOTAIR manipulates apoptosis-related proteins via miR-34a The expression of apoptosis-related proteins including Bax, Bcl-2 and cleaved caspase-3 in HepG2/Taxol and SMMC7721/Taxol cells with different treatments were detected by western blotting. The means of three independent biological replicates are shown; error bars indicate the SD. *P<0.05 vs. different controls.

The expression levels of apoptosis-related proteins including BAX, Bcl-2 and cleaved caspase-3 were evaluated in the two Taxol-resistant cell lines with Western blot. As displayed in Figure 6, cleaved caspase-3 and Bax were significantly up-regulated by si-HOTAIR transfection or/and Taxol treatment, while Bcl-2 level exhibited opposite trend. Low-expressed HOTAIR augmented the toxicity of Taxol, while the toxicity caused by si-HOTAIR transfection or/and Taxol treatment was weakened by anti-miR-34a transfection. Taken together, HOTAIR knockdown enhances apoptosis induced by Taxol in HepG2/Taxol and SMMC7721/Taxol cells via interacting with miR-34a.

### HOTAIR modulates Akt phosphorylation and Wnt/β-catenin signaling via miR-34a

**Figure 7:**
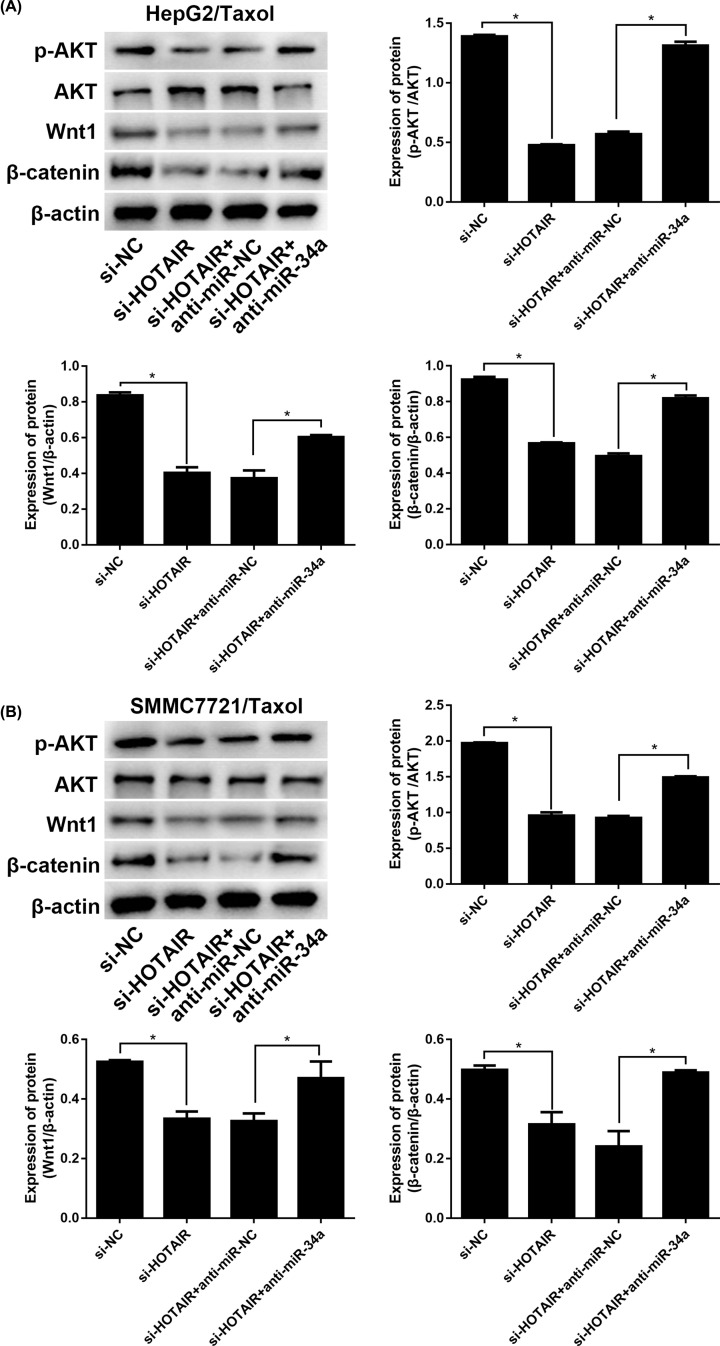
HOTAIR manipulates the Akt phosphorylation and Wnt/βcatenin signaling via miR-34a The protein levels of p-Akt, Akt, Wnt1, and β-catenin in HepG2/Taxol and SMMC7721/Taxol cells with different treatments were evaluated by Western blotting. The means of three independent biological replicates are shown; error bars indicate the SD. *P<0.05 vs. different controls.

To identify the related in-depth mechanism of HOTAIR, Akt phosphorylation and Wnt/β-catenin signaling were evaluated. The data in Figure 7A,B indicated that the protein levels of Wnt1 and β-catenin were significantly down-regulated due to HOTAIR knockdown, as well as the ratio of p-Akt/Akt. Besides, after adding miR-34a inhibitor, the functions of si-HOTAIR transfection were reversed. All the results revealed that HOTAIR modulated Akt phosphorylation and Wnt/β-catenin signaling via miR-34a.

## Discussion

Although anticancer drugs, for instance Taxol, have been widely applied in the treatment of hepatocellular carcinoma, chemoresistance is still an important therapeutic difficulty and its molecular mechanisms are poorly known [30]. More and more studies have shown that the abnormal expression of HOTAIR is closely related to the different biological behaviors of human tumors and has gradually become a research hotspot in the field of cancer. The biological role of HOTAIR is to regulate the downstream target oncogenes by recruiting a series of complex protein complexes. Over-expression of HOTAIR has been shown to play a promising role in the occurrence and predictive prognosis of a variety of epithelial-derived malignancies including pancreatic cancer, non-small cell lung cancer, and glioma [22,31–33]. Recent studies have also found that the expression level of HOTAIR is not only related to postoperative prognosis evaluation of liver cancer patients, but also related to its resistance to platinum preparations and doxorubicin and invasion and metastasis of liver cancer cells [14]. In the current research, HOTAIR was highly up-regulated in HepG2/Taxol and SMMC7721/Taxol cells, compared with their normal HepG2 and SMMC7721 cells.

Additionally, miRNAs also have diverse functions in a variety of diseases, including cancer, and are primarily involved in inhibiting gene expression at the post-transcriptional level, which in turn regulates cell growth, proliferation, cell cycle regulation, differentiation, and apoptosis. With luciferase reporter assay, miR-34a was validated as the binding target of HOTAIR. Recent studies have also found that miR-34a can down-regulate Wnt/β-catenin signaling pathway, epidermal growth factor receptor, caspase family-associated protein and EMT in various cancer tissues and inhibit cell proliferation, promote apoptosis and inhibit metastasis. In the present study, HOTAIR and miR-34a was demonstrated with a negative correlation HCC/Taxol cells. MiR-34a level was highly up-regulated in HOTAIR-knockdown HepG2/Taxol and SMMC7721/Taxol cells. HOTAIR knockdown could suppress invasion of in HepG2/Taxol and SMMC7721/Taxol cells. Besides, miR-34a inhibitors impaired the function of HOTAIR-knockdown on the Taxol-resistance in HepG2/Taxol and SMMC7721/Taxol cells, indicating that the results rely on the modulation of miR-34a.

Akt phosphorylation plays a vital role in the biology of cancers, for instance, tumorigenesis, metastasis, and resistance to traditional chemotherapeutic agents [34]. The addition of Taxol was shown to increase Akt activation, in accordance with less cell death [35]. Induction of Akt activation by cisplatin, as well, was in charge of cisplatin-resistance observed [36]. Nevertheless, the exact mechanism by which Akt overactivation leads to drug-resistance remains unknown [37]. It is also reported that cell proliferation and tumor progression in glioma stem cells were inhibited by miRNA-34a by targeting Akt and Wnt signaling. However, whether HOTAIR could contribute Taxol-resistance of HCC cells via activating AKT phosphorylation by regulating miR-34a was still unknown. The present study confirmed this conjecture.

## Conclusion

In conclusion, the function of HOTAIR in Taxol-resistance and its related potential mechanism in hepatocellular carcinoma were revealed. It was showed that HOTAIR and miR-34a were abnormally expressed in HepG2/Taxol and SMMC7721/Taxol cells. HOTAIR knockdown suppresses invasion and promotes apoptosis of in HepG2/Taxol and SMMC7721/Taxol cells through up-regulating miR-34a, while down-regulation of miR-34a had an opposite effect on reversing Taxol resistance, indicating that HOTAIR is miR-34a-dependent. Besides, HOTAIR accommodates the Akt phosphorylation and Wnt/β-catenin signaling. The present study affords important insight for the identification and characterization of a new molecular target and a marker for Taxol-resistance of hepatocellular carcinoma therapy.

## Abbreviations

HCC: Hepatocellular carcinoma
HOTAIR: HOX transcript antisense RNA
lncRNA: long noncoding RNAs
miRNA: microRNA
qPCR: quantitative PCR
