# Correction: LncRNA HOTAIR contributes Taxol-resistance of hepatocellular carcinoma cells via activating AKT phosphorylation by down-regulating miR-34a

**DOI:** 10.1042/BSR-2020-1627_COR

**Published:** 2021-08-02

**Keywords:** apoptosis, Taxol-resistance

The authors of the original article “LncRNA HOTAIR contributes Taxol-resistance of hepatocellular carcinoma cells via activating AKT phosphorylation by down-regulating miR-34a” (*Biosci Rep* (2020) **40**(7), https://doi.org/10.1042/BSR20201627) would like to correct Figure 4C, as the image used in the anti-NC group had been taken from the si-NC group of images in error during the figure preparation for their manuscript's submission to the journal. The correct image from the anti-NC group has replaced this error in the updated Figure 4 present in this Correction.

**Figure 4:**
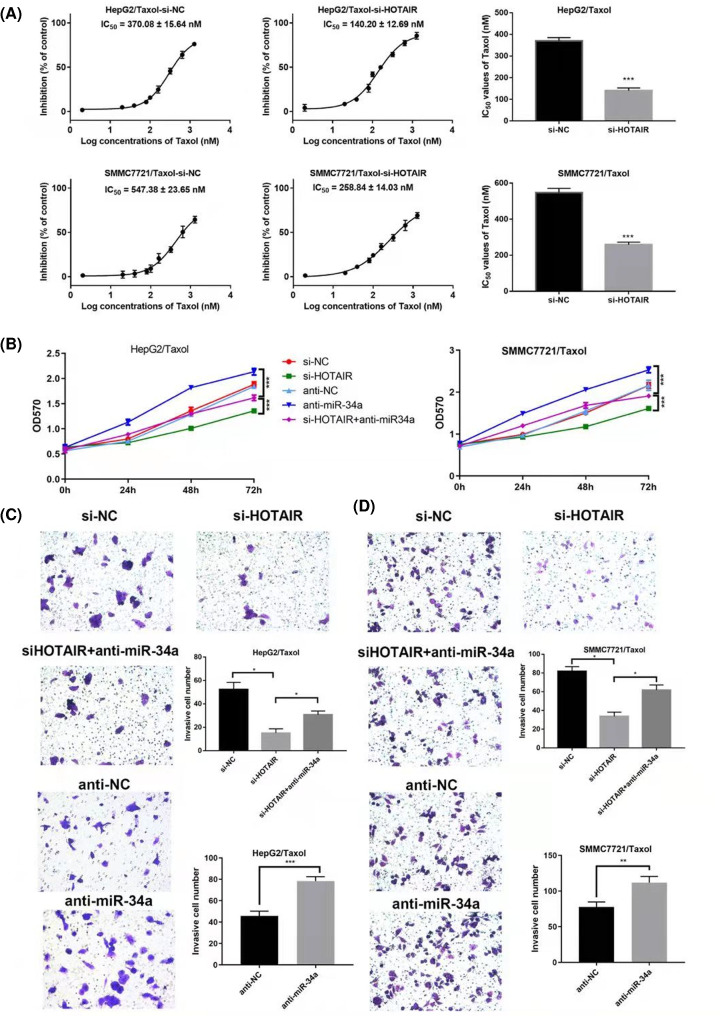
HOTAIR knockdown suppresses proliferation and invasion of HepG2/Taxol and SMMC7721/Taxol cells through up-regulating miR-34a (**A**) Proliferation rates and IC50 values of HepG2/Taxol or SMMC7721/Taxol after transfections. (**B**) The proliferation curves after transfections in HepG2/Taxol or SMMC7721/Taxol cells. Typical images of the invasion assays: (**C**) HepG2/Taxol cells treated with si-NC; si-HOTAIR; si-HOTAIR+anti-miR-34a; anti-NC; anti-miR-34a; (**D**) SMMC7721/Taxol cells treated with si-NC; si-HOTAIR; si-HOTAIR+anti-miR-34a; anti-NC; anti-miR-34a. The means of three independent biological replicates are shown; error bars indicate the SD; *P<0.05, **P<0.01, ***P<0.001.

